# Protective effect of a Protein Epitope Mimetic CCR10 antagonist, POL7085, in a model of allergic eosinophilic airway inflammation

**DOI:** 10.1186/s12931-015-0231-5

**Published:** 2015-06-27

**Authors:** François Daubeuf, Françoise Jung, Garry J. Douglas, Eric Chevalier, Nelly Frossard

**Affiliations:** Laboratoire d’Innovation Thérapeutique, Unité Mixte de Recherche 7200, Centre National de la Recherche Scientifique-Université de Strasbourg and LabEx Medalis, Faculté de Pharmacie, 74, route du Rhin, 67400 Illkirch, France; Polyphor Ltd, Hegenheimermattweg 125, CH-4123 Allschwil, Switzerland

**Keywords:** CCR10, CCL28, Asthma, Airway hyperresponsiveness, Inflammation, Mouse, Allergen, Ovalbumin, Eosinophilia

## Abstract

**Background:**

Potential involvement of the CCR10/CCL28 axis was recently reported in murine models of allergic asthma. If confirmed, blockade of the CCR10 receptor would represent an alternative to current asthma therapies. We evaluated the effect of a novel Protein Epitope Mimetic CCR10 antagonist, POL7085, in a murine model of allergic eosinophilic airway inflammation.

**Methods:**

Mice were sensitized and challenged to ovalbumin. POL7085, a CCR10 antagonist (7.5 and 15 mg/kg), dexamethasone (1 mg/kg) or vehicle were administered intranasally once daily 1h before each allergen challenge. On day 21, airway hyperresponsiveness, bronchoalveolar lavage inflammatory cells and Th2 cytokines, and lung tissue mucus and collagen were measured.

**Results:**

Allergen challenge induced airway hyperresponsiveness in vehicle-treated animals as measured by whole body barometric plethysmography, and eosinophilia in bronchoalveolar lavage. POL7085 dose-dependently and significantly decreased airway hyperresponsiveness (34 ± 16 %) and eosinophil numbers in bronchoalveolar lavage (66 ± 6 %). In addition, the highest dose of POL7085 used significantly inhibited lung IL-4 (85 ± 4 %), IL-5 (87 ± 2 %) and IL-13 (190 ± 19 %) levels, and lung collagen (43 ± 11 %).

**Conclusions:**

The Protein Epitope Mimetic CCR10 antagonist, POL7085, significantly and dose-dependently decreased allergen-induced airway hyperresponsiveness and airway inflammation after once daily local treatment. Our data give strong support for further investigations with CCR10 antagonists in asthmatic disease.

**Electronic supplementary material:**

The online version of this article (doi:10.1186/s12931-015-0231-5) contains supplementary material, which is available to authorized users.

## Background

Allergic asthma is a chronic inflammatory disease of the airways characterized by intermittent episodes of wheezing and coughing. The clinical manifestation of asthma is caused by an inappropriate response to inhaled allergens, with reversible airway obstruction, airway hyperresponsiveness (AHR), infiltration of inflammatory cells into the bronchial tissue, mucus metaplasia, allergen-specific IgE production, and over expression of Th2-driven cytokines and chemokines such as IL-5, CCL5 and CCL11. Recently, a possible role for the CCL28/CCR10 axis in airway disease was postulated [1–4].

CCR10/GPR2 [5, 6] and its two cognate ligands, CCL27 and CCL28 have been implicated in the regulation of epithelial immunity and related diseases [7, 8]. High expression of CCR10 has been noted in epithelia of skin, small intestine, colon, salivary glands, mammary glands, and fetal lung [9–11]. In addition, other cell types have been reported to express high levels of CCR10, such as melanocytes, dermal fibroblasts, dermal microvascular endothelial cells and skin T cells [6, 12], sub-populations of immune cells such as IgE-secreting B cells [9, 13] and IgA-secreting plasma cells in mucosal tissues [7].

Interestingly, the expression of the two ligands for CCR10 is non redundant. CCL27 is expressed in skin by keratinocytes [12] whereas CCL28 is predominantly expressed by epithelial cells of various mucosal tissues such as the airway epithelium, salivary glands, mammary glands, colon, but also by eosinophils and T cells [9–11]. The two chemokines also differ in their selectivity for receptors, since CCL27 uniquely activates CCR10, whereas CCL28 activates both CCR10 and CCR3. CCL28 mediates *in vitro* T and B cell migration through CCR10 [9, 13] whereas it induces migration of human blood eosinophils in a CCR3-dependent fashion [10]. CCL28 is also thought to play a role in mucosal immunity because of its intrinsic potent bactericidal activity [11, 14].

The involvement of CCL28 and its receptors CCR3 and CCR10 has been implicated in inflammatory lung diseases including asthma. CCL28 mRNA expression is reported in both normal and asthmatic lung tissues [9]. In A549 airway epithelial cells, CCL28 expression is increased upon stimulation with the pro-inflammatory IL-1β and TNF-α [3] and IL-17A [13]. Furthermore, CCL28 mRNA is substantially higher in biopsy tissue and sputum samples from asthmatics compared to healthy volunteers [3], and CCL28 protein concentrations in asthmatic sputum correlates with IL-17A, CCR10 and CCR3 mRNA expression [13].

A role for CCL28 and CCR10 in various models of lung inflammation in mice has also been suggested. CCL28 expression was increased in the lung of mice sensitized and challenged with cockroach antigen, and was associated with increased lung expression of CCR3 but not CCR10, and treatment with CCL28 antiserum reduces peribronchial eosinophilia and AHR [2]. By contrast, in a model of Th2 allergic airway inflammation in mice sensitized and challenged with ovalbumin (OVA), inflammation was accompanied both by upregulation of CCL28 [1], and increased number of CCR10^+^ cells [1, 15].

**Fig. 1:**
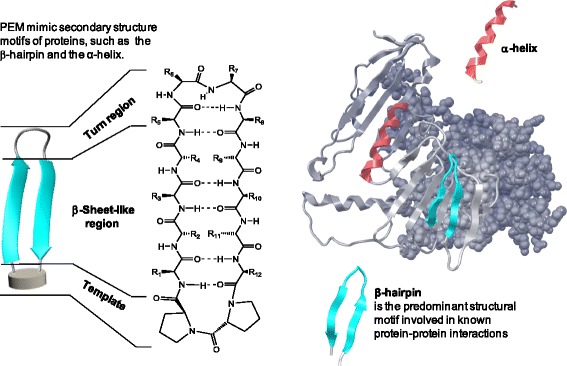
Representative structure of Protein Epitope Mimetic (PEM) molecules

Taken together, this evidence suggests relevance for the CCR10/CCL28 axis in respiratory diseases and, in particular asthma. We therefore explored the pathophysiological role of CCR10 in a murine model of allergic asthma by use of a novel CCR10 antagonist developed from Protein Epitope Mimetic (PEM) technology (Fig. 1). PEM are medium sized, fully synthetic cyclic peptide-like molecules that mimic the two most relevant secondary structure motifs involved in protein-protein interactions, ß-hairpins and α-helices [16]. Optimization of a primary hit family, identified by screening a GPCR-focused subset of the PEMfinder® library against the human CCR10 receptor, led to the discovery of POL7085 a potent CCR10 antagonist [17]. We therefore evaluated whether antagonizing the CCR10 receptor with POL7085 could reduce the inflammation-related effects of allergen challenge in a mouse model of OVA-induced allergic eosinophilic airway inflammation.

## Methods

### Mice

Nine-week-old male Balb/c mice were purchased from Janvier Laboratories. Animals were acclimatized under controlled environmental conditions for at least one week before use. Animals were maintained under controlled environmental conditions in conventional husbandry at constant temperature (20 ± 2 °C) with a relative humidity (50 ± 10 %) and 12 h/12 h light–dark cycle (lighting 07:00–19:00). Mice were housed in polycarbonate cages with four mice per cage (PCT2L12SHT) with bedding made from spruce wood chips (Safe). The room air was ventilated at ten air changes per hour according to the European recommendations. Food (standard diet) and tap water were available *ad libitum*. Cages with bedding were changed weekly. Animal experimentation was conducted with the approval of the Local Ethics Committee of the Strasbourg University (Comité Régional d’Ethique en Matière d’Expérimentation Animale de Strasbourg).

### Synthesis

POL7085 was produced by fluorenylmethoxycarbonyl solid-phase based synthesis on highly acid labile chlorotrityl chloride resin (100–200 mesh, Novabiochem, 01-64-0114) and backbone cyclised [17].

### Calcium flux assays

Calcium mobilization assays were performed in Chem-1 cells expressing the human CCR10 receptor (hCCR10; Millipore) loaded with Fluo-4 or in CHO-K1 cells expressing the murine CCR10 receptor (mCCR10) in an apo-aequorin assay (Euroscreen). CCL27 and CCL28 were used at their respective EC_80_ concentrations (70 nM for CCL27 and 22 nM for CCL28).

### Allergen sensitization and challenge

Groups of 6 to 12 Balb/c mice were sensitized i.p. on days 0 and 7 with 2 mg/kg chicken egg albumin (OVA; Sigma-Aldrich, A5503) adsorbed on 80 mg/kg aluminium hydroxide (Al(OH)_3_; Sigma-Aldrich) in saline. Under i.p. ketamine and xylazine anaesthesia (respectively: Imalgene®, Merial, 50 mg/kg; Rompun®, Bayer, 3.33 mg/kg), sensitized mice were challenged daily on days 18–21 by intranasal (i.n.) instillation of 400 μg/kg OVA in saline, or saline alone for controls (1 mL/kg) [18]. Control animals were sensitized with OVA and challenged with saline.

### Treatment with POL7085 and dexamethasone

POL7085 was used at the doses of 7.5 and 15 mg/kg, and dexamethasone (DEX) at the dose of 1 mg/kg. Compounds were solubilised in 10 % (2-hydroxypropyl)-β-cyclodextrin (CDX; Sigma-Aldrich, C0926), and administered i.n. one hour before each OVA challenge. Control animals received equivalent volumes (1 mL/kg) of vehicle.

### Measurement of airway responsiveness

Airway responsiveness to aerosolized methacholine (MCh; Sigma-Aldrich, A2251) was measured in conscious mice on day 22 (24 h after the last OVA challenge) by whole body barometric plethysmography (Emka Technologies). Mice were acclimatized in the plethysmograph chamber for 30 min until a stable baseline was obtained, and then exposed to aerosolized saline for 30 s as a control. Mice were then challenged every 20 min with aerosolized MCh at increasing concentrations (0.05, 0.1, 0.2 and 0.3 M) for 30 s, and the enhanced pause (PenH) was recorded for 5 min and used as an index of airway obstruction [19, 20].

### Total and differential cell counts

Bronchoalveolar lavage (BAL) was performed 24 h after the last OVA challenge as previously described [21]. Mice were anaesthetized i.p. (ketamine 150 mg/kg, xylazine 10 mg/kg). A plastic cannula was inserted in the exposed trachea, and airways were washed with 0.5 mL of 0.9 % NaCl (saline) injected with a 1 mL syringe. This procedure was performed ten times. The initial concentrated supernatant of the two first lavages (2 × 0.5 mL administered, approx. 0.5 mL recovered) was collected for cytokine measurements. The remainder of the BALF was centrifuged (600 *g* for 5 min, 4 °C), and cell pellets pooled. After lysis of erythrocytes with distilled water followed by osmotic re-equilibration and centrifugation, the cell pellet was suspended in 500 μL of saline and total cell counted on a haemocytometer chamber (Neubauer, PRECISS®). Cells were cytocentrifuged at 700 rpm for 10 min (Cytospin 4, Thermo Fischer Scientific), and stained with Diff-Quick® (Merck) for differential cell counts performed on at least 400 cells.

### Enzyme-Linked Immunosorbent Assays (ELISA)

IL-5, IFN-γ (BD Pharmingen), CCL28 (R&D system) ELISA kits and Th17 Milliplex® (Millipore) were used according to the manufacturer’s instructions. For BALF recovered 24 h after the last OVA challenge, IL-5 and IFN-γ were quantified by ELISA. For lung homogenate, concentrations of IL-4, IL-5, IL-13, IL-17a and IL-17f were quantified by Milliplex® in the supernatants obtained after lung tissue was homogenised with an UltraTurax® and centrifuged at 10,000 *g* for 10 min at 4 °C.

Plasma IgE levels were determined by ELISA. Microtiter plates were coated overnight at 4 °C with the capture antibody, anti-mouse IgE at 2 μg/well in carbonate buffer, pH 9.5 (BD Pharmingen, clone R35-118). OVA-specific IgE levels were measured using ovalbumin-HRP (BUF048, Abdserotec) revealed with TMB reagent set (Pharmingen).

### Colorimetric assay of mucus and collagen

Mucus was measured in lung homogenate by a mucus colorimetric assay: 100 μL samples were mixed with 20 μL acid mucus reagent (0.2 % periodic acid) incubated for 1 h at 37 °C, then 20 μL mucus dye reagent (pararosaniline 1 %, 10 N hydrochloric acid 1 %) was added and samples were incubated for a further 30 min at 37 °C, before optical density measurement at 555 nm.

Collagen in the lung was measured using a collagen colorimetric assay: 10 μL lung homogenate samples were added to 200 μL collagen dye reagent (Sirius red 0.017 %, and picric acid 0.8 % in absolute ethanol), vortexed for 30 min at 200 rpm at RT, centrifuged at 2400 *g* for 20 min at 4 °C. Pellets were solubilised with 200 μL alkali collagen reagent (absolute ethanol 20 %, sodium dodecyl sulphate 1.3 %, 2 N sodium hydroxide 12 %), and optical density measured at 540 nm.

### Histological analysis

Lung tissues were fixed in 4 % paraformaldehyde and paraffin-embedded. 6 μm sections were cut, mounted on Superfrost glass slides (Fischer Scientific) and stained with haematoxylin-eosin for light microscopy morphological analysis or periodic acid-Schiff (PAS) staining to determine the extent of mucus production. The area of epithelial cells containing the mucus were measured and expressed relative to the total bronchus perimeter.

### Immunohistochemistry of CCR10

Sections (3 μm) of lung tissue were cut, mounted, placed in Sequenza® immunostaining with Coverplate™ (Thermo Scientific Shandon), and antigen retrieval was performed with proteinase K in Tris-EDTA buffer (pH 8; Fluka) for 35 min at 37 °C. The endogenous enzyme was blocked with Dual Endogenous Enzyme Block (Dako Cytomation) and slides were incubated overnight at 4 °C with the primary antibody 1/1000 (goat anti-mouse CCR10 antibody; Capralogics), followed by 30 min at RT with the secondary rabbit anti-goat antibody (Vector) and 30 min at RT with extravidin peroxidase (Sigma-Aldrich). Staining was revealed with a peroxidase substrate (DAB) and slides counterstained with Fastblue and images were acquired with a DP72 camera coupled to cellSens® software (Olympus).

### Real time quantitative PCR (RT-qPCR) analysis

All procedures were performed according to the manufacturer’s instructions. Lung tissue was homogenised in a Fastprep®, and total RNA extracted with Tri-Reagent® (1 mL/left lung; Euromedex) and purified with mini RNeasy® Kit (Qiagen). RNA pellets were suspended in nuclease-free water (DEPC). RNA concentration and quality were determined in each preparation with a Nanodrop® spectrophotometer.

Reverse transcription was performed on 10 μg/mL of total RNA by the High Capacity cDNA Archive kit® (Life Technologies™) for 10 min at 25 °C and 2 h at 37 °C, and cDNAs were stored at −80 °C until use. Quantitative PCR was performed by real-time fluorescent PCR with TaqMan® Gene Expression Assay in 96 well plate format (Life Technologies™, ABI7000) using TaqMan® Gene Expression Assay primers (Life Technologies™ for HPRT1 (Mm01545399_m1), CCR10 (Mm01292449_m1), CCL28 (Mm00445039_m1) and CCR3 (Mm00515543_s1). To analyse the low expression of CCR10, CCL28 and CCR3, a preamplification was performed with TaqMan® PreAmp Master Mix (Life Technologies™) and TaqMan® Gene Expression Assay (10 min at 95 °C followed by 14 cycles: 15 s at 95 °C and 4 min at 60 °C). Preamplified cDNAs were stored at −80 °C until use and diluted 1/20 with 1X TE buffer for use. Reactions were conducted in a 25 μL final volume containing 12.5 μL TaqMan® Universal PCR Master Mix (Life Technologies™), 5 μL cDNA, 1.25 μL TaqMan® Gene Expression assay and 6.25 μL DEPC water for 10 min at 95 °C followed by 40 cycles: 15 s at 95 °C and 1 min at 60 °C. Results are expressed as cycle thresholds (Ct) that reflect the cycle number at which the fluorescence curve generated within a reaction crossed the amplification threshold. CCR10, CCL28 and CCR3 gene expressions were normalized to the expression of the domestic control gene HPRT1 (ΔCt = Ct_gene_−Ct_HPRT1_). Following normalization, gene expression compared to the mean expression in the control group (ΔΔCt−ΔCt_sample_−ΔCt_control_) and transformed (2^ΔΔCt^) to obtain the difference in numbers of copies compared to the control group.

### Plasma and tissue measurements of POL7085

Plasma and lung tissue concentrations of POL7085 after i.n. administration were determined in an independent experiment. POL7085 was dissolved in CDX and administered i.n. (12.5 μL per nostril) at 15 mg/kg in BALB/cBy mice anaesthetised i.p. with ketamine and xylazine. Under terminal anaesthesia (sodium pentobarbital, 60 mg/kg i.p.), blood was drawn from the vena cava at 5 min, 1 h, 4 h, 8 h and 24 h post-administration, anticoagulated with lithium heparin, centrifuged at 500 *g* for 10 min and the resulting plasma was stored at −20 °C. Lung lobes were dissected out, weighed and stored frozen at −20 °C until analysis. Concentrations of POL7085 in plasma and lung homogenate supernatants were determined using a high performance liquid chromatography coupled to mass spectrometry detection (UPLC-4000 Q Trap, Applied Biosystems/MDS Sciex).

### Statistical analysis

Data are presented as means ± standard errors of the mean (SEM). Statistical significance between vehicle and POL7085 treatment groups or vehicle and DEX treatment groups in the saline- or OVA-challenged mice was determined using Prism software (GraphPad) by a two-way ANOVA and Bonferroni post-hoc test for PenH, a one-way ANOVA followed by Bonferroni post-hoc test for BAL cellular profiles, and a one-way ANOVA followed by Tukey post-test for biochemical measurements. Data were considered significantly different when *p* < 0.05.

## Results

### Characteristics of POL7085

POL7085 is a potent and selective antagonist of human and murine CCR10 receptors. POL7085 inhibited calcium flux induced by both CCL27 (IC_50_ = 42 and 58 nM for hCCR10 and mCCR10, respectively) and CCL28 (IC_50_ = 45 nM; hCCR10). When tested at concentrations up to 10 μM, the selectivity of POL7085 was more than 200-fold against a panel of 57 targets, including all chemokine receptors, GPCRs, cytokine receptors, ion channels and enzymes [see Additional file 1].

### Lung and plasma concentrations of POL7085

**Fig. 2:**
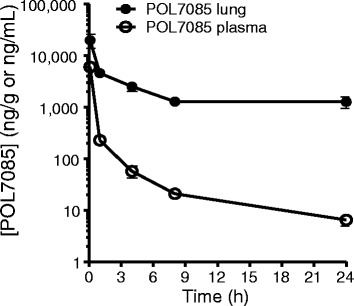
Lung and plasma concentrations of POL7085 over time following i.n. administration of POL7085 at a dose of 15 mg/kg in mice. Points are means ± SEM values for *n* = 3 mice per data point

Five min after i.n. administration of POL7085 at 15 mg/kg, lung tissue concentration was 19.9 μg/g, decreasing to 4.5 μg/g at 1 h, followed by a slower absorption with concentration at 24 h of 1.3 μg/g and limited systemic levels (7 ng/mL). These levels are compatible with once daily treatment with POL7085 with lung exposures above the IC_50_ for 24 h (Fig. 2).

In the 21-day model of allergic eosinophilic airway inflammation, similar mean concentrations of POL7085 were found in lung (1.6 μg/g) and plasma (1.8 ng/mL) 24 h after the last i.n. administration of POL7085 (not shown).

### Differential cell counts in BAL

**Fig. 3:**
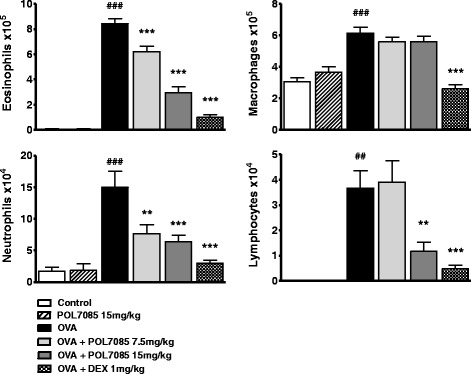
Effect of intranasal POL7085 on BAL inflammatory cells 24 h after the last challenge. Total numbers of eosinophils, macrophages, neutrophils and lymphocytes recovered in BALF in OVA-sensitized mice challenged with OVA or saline. Columns represent group means and bars are SEM values (*n* = 6 (saline) or 12 (OVA) per group). ^###^ *P* ≤ 0.001 *vs* control and **P ≤ 0.01, ***P ≤ 0.001 *vs* OVA

Compared to vehicle, POL7085 alone (15 mg/kg) had no effect on the number of cells recovered in BAL in non-sensitized animals (Fig. 3). Twenty-four hours after the last OVA challenge, there was a significant increase of inflammatory cells in BALF in OVA compared to control groups (*p* < 0.001). POL7085 (15 mg/kg) significantly inhibited eosinophil, neutrophil and lymphocyte recruitment by 65, 64 and 68 %, respectively (*p* < 0.001) (Fig. 3). Similarly, the reference compound DEX significantly decreased BAL eosinophil, neutrophil and lymphocyte numbers (87, 90 and 86 %, respectively; *p* < 0.001).

### Cytokines in BAL and lung homogenate and OVA-specific IgE levels in plasma

**Fig. 4:**
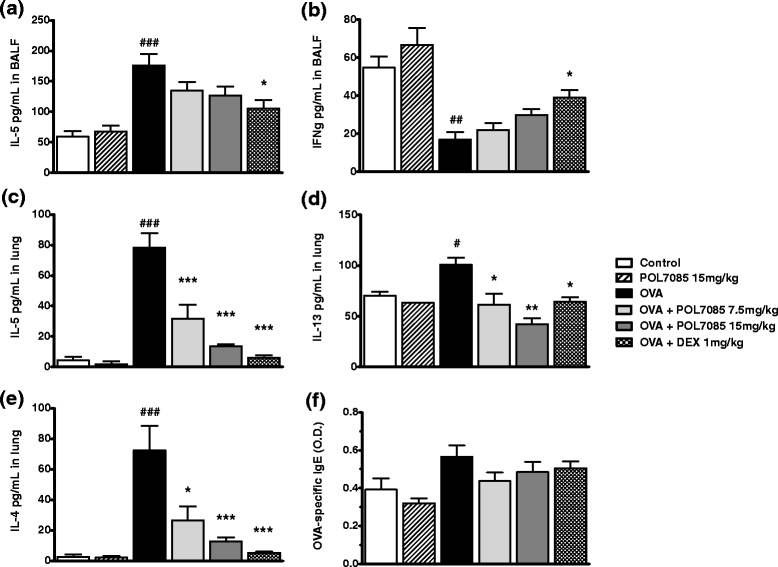
Effect of POL7085 on BAL and lung cytokine concentrations and OVA-specific IgE levels in plasma. **a**,**b** BAL IL-5 and IFN-γ quantified by ELISA. **c**-**e** Lung tissue IL-5, IL-13 and IL-4 quantified by Milliplex®. **f** Plasma OVA-specific IgE levels quantified by ELISA. Columns represent group means and bars are SEM values for *n* = 6 or 12 per group (**a**,**b**) or *n* = 3–6 per group (**c**-**f**). ^#^ *P* ≤ 0.05, ^##^ *P* ≤ 0.01, ^###^ *P* ≤ 0.001 *vs* control; *P ≤ 0.05, ***P ≤ 0.001 *vs* OVA

OVA significantly increased the levels of IL-5 in BAL (*p* < 0.001); these levels were reduced by POL7085, although not significantly (Fig. 4a). IFN-γ levels were significantly decreased by OVA treatment in BAL (*p* < 0.01) (Fig. 4b), and slightly but non-significantly restored by POL7085.

OVA challenge significantly increased the levels of IL-4 (*p* < 0.001), IL-5 (*p* < 0.001) and IL-13 (*p* < 0.05) in lung homogenate (Fig. 4c-e). These levels were dose-dependently and significantly reduced by POL7085 (*p* < 0.001, *p* < 0.05 and *p* < 0.001, respectively) (Fig. 4c-e). IL-17a and IL-17f were not detectable (data not shown). The levels of detectable cytokines were also significantly decreased by the reference compound DEX in both BAL and lung homogenate.

For IgE levels, all animals were sensitized in this study and were challenged with OVA or saline on D18-21. OVA-specific IgE levels did not increase significantly 24h after the last OVA challenge on D22, and a slight decrease, although non significant, was noted with POL7085 or DEX (Fig. 4f).

### Expression of CCL28 and its receptors in the lung

**Fig. 5:**
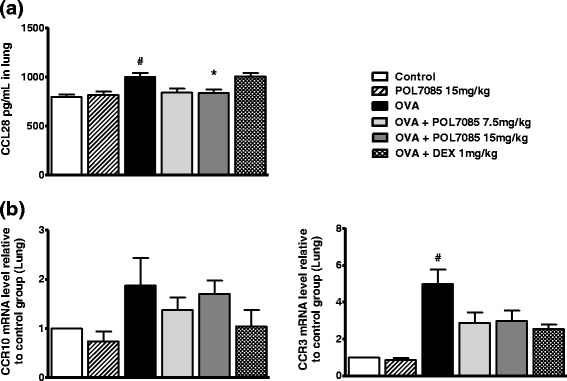
Effect of POL7085 on lung CCL28 concentration and its receptors mRNA. (**a**) Lung tissue CCL28 levels quantified by ELISA. (**b**) Lung tissue CCR10 and CCR3 mRNA levels relative to vehicle-treated, saline challenged control group (2^ΔΔCt^) quantified by RT-qPCR. Columns represent group means and bars are SEM values for *n* = 6 or 12 per group (a) or *n* = 2–6 per group (b). ^#^ *P* ≤ 0.05 *vs* control; *P ≤ 0.05 *vs* OVA

CCL28 levels were significantly increased by approximately 25 % in lung (*p* < 0.05) following OVA challenge. Treatment with POL7085 reduced lung CCL28 concentrations to baseline levels (Fig. 5a, *p* < 0.05). By contrast, CCL28 chemokine mRNA levels were not significantly modified in any group at 24 h after the last OVA challenge (not shown). CCL28 levels in BAL were highly variable and inconsistent (not shown). The reference compound DEX had no effect on CCL28 levels.

Expression of the two CCL28 receptors (CCR10 and CCR3) mRNA was quantified in the lung as relative to that of the control group (2^ΔΔCt^) (Fig. 5b). CCR10 mRNA levels were highly variable in animals sensitized and challenged with OVA, and it was not possible to determine whether treatment by POL7085 or DEX had any effect. The 5-fold increase in lung CCR3 mRNA expression induced by OVA challenge (*p* < 0.05) was reduced in mice treated with POL7085 (by 42 %) although reduction did not reach significance. The reference compound DEX also showed a non-significant inhibition (45 %).

### Remodelling: mucus and collagen colorimetric assays

**Fig. 6:**
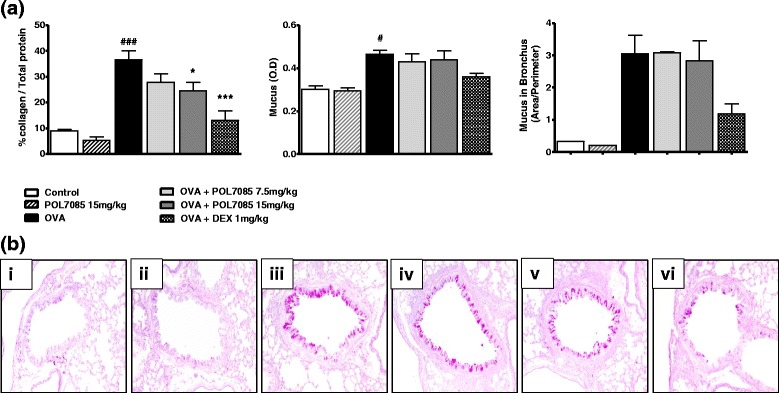
Effect of POL7085 on lung mucus and collagen levels. **a** Columns represent group means and bars are SEM values (*n* = 3–6 per group). ^#^ *P* ≤ 0.05, ^###^ *P* ≤ 0.001 *vs* control and *P ≤ 0.05, ***P ≤ 0.001 *vs* OVA. **b** Representative lung tissue sections (3 μm) showing mucus stain with periodic acid Schiff, 24 h after the last saline or OVA challenge (magnification x100). Mice received the following treatments: (i) vehicle, saline challenge, (ii) POL7085 15 mg/kg, saline challenge, (iii) vehicle, OVA challenge, (iv) POL7085 7.5 mg/kg, OVA challenge, (v) POL7085 15 mg/kg, OVA challenge, (vi) DEX 1 mg/kg, OVA challenge

Mucus and collagen were measured in lung homogenates. OVA challenge increased both mucus (1.4-fold; *p* < 0.05) and collagen (3.7-fold; *p* < 0.001) in the lung (Fig. 6a). Treatment with POL7085 had no effect on mucus, a result confirmed by histology analysis (PAS; Fig. 6b), but showed significant reduction of collagen levels (40 %; *p* < 0.05) (Fig. 6a). The reference compound DEX showed a trend towards reduction of mucus levels (ns), and a significant reduction of collagen levels (64 %; *p* < 0.001).

### Airway responsiveness

**Fig. 7:**
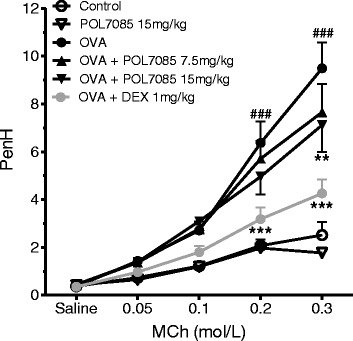
Effect of POL7085 on AHR. Airway reactivity to increasing concentrations of inhaled MCh (0.05-0.3 M) was measured by whole body plethysmography in unrestrained conscious mice. Symbols represent group means and bars are SEM values (*n* = 6 to 12 per group). ^###^ *P* ≤ 0.001 *vs* control and *P ≤ 0.05, ***P ≤ 0.001 *vs* OVA

Whole body barometric plethysmography was used as an index of airway obstruction. Twenty-four hours after the last OVA challenge, inhaled MCh induced a significant increase in Penh values as compared to saline-treated mice (3.7-fold at 0.3 M MCh; *p* < 0.001). POL7085 had no effect in saline-challenged animals, but significantly reduced airway obstruction in OVA-challenged mice treated with POL7085 at the highest dose (15 mg/kg; 30 %; *p* < 0.05; Fig. 7). As expected, the reference compound DEX also significantly decreased airway responses to MCh (*p* < 0.001).

### Histology

**Fig. 8:**
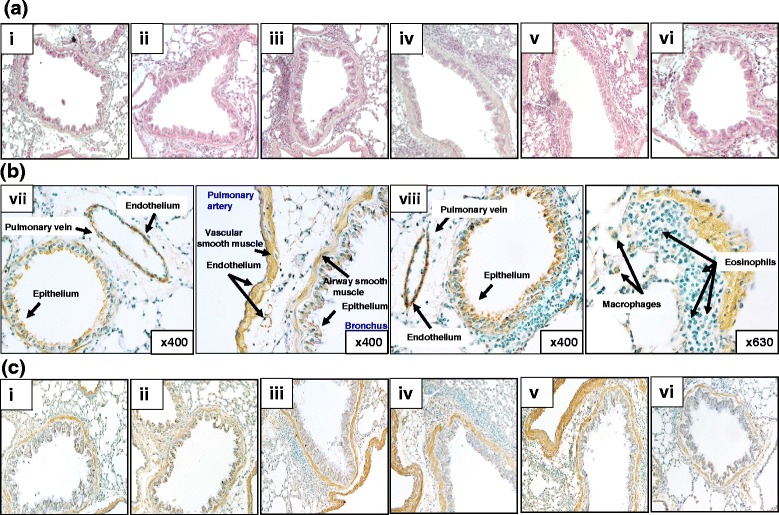
Representative lung tissue sections (3 μm) showing peribronchial inflammatory infiltrates 24 h after the last saline or OVA challenge. **a** Staining with haematoxylin & eosin (magnification x100). **b** Immunolabelling of CCR10 in mice challenged with saline (vii) or OVA (viii). CCR10 is expressed on bronchial and arterial smooth muscle, epithelial and endothelial cells, and occasional inflammatory cells (magnification x400 or x630). **c** Immunolabelling of CCR10 on lung sections (magnification x200). Mice received the following treatments: (i) vehicle, saline challenge, (ii) POL7085 15 mg/kg, saline challenge, (iii) vehicle, OVA challenge, (iv) POL7085 7.5 mg/kg, OVA challenge, (v) POL7085 15 mg/kg, OVA challenge, (vi) DEX 1 mg/kg, OVA challenge

Lungs of mice challenged with saline and treated with either vehicle or POL7085 showed no sign of inflammation. Lung tissues of OVA-challenged mice were characterized by marked peribronchial and perivascular inflammatory cell infiltration (Fig. 8a and b), mostly composed of eosinophils and macrophages with lower numbers of neutrophils and lymphocytes. POL7085- (15 mg/kg) and DEX-treated mice exhibited a substantial reduction of these cellular infiltrates into the lung (Fig. 8a, iv and v).

### Immunohistochemistry (IHC) of CCR10 in the lung

IHC was performed on paraffin-embedded lung sections, with a goat anti-mouse CCR10 antibody. In control mice challenged with saline, CCR10 immunoreactivity was present on airway epithelial and vascular endothelial cells as well as on smooth muscle of the airways and pulmonary artery (Fig. 8b and c). Additionally, in OVA-challenged mice, occasional inflammatory cells, including macrophages, were stained positively for CCR10 (Fig. 8b viii). Treatment with POL7085 did not affect the distribution of CCR10 observed in lung tissue (Fig. 8c iv and v).

## Discussion

Our study reports the effect of the novel selective PEM CCR10 antagonist POL7085 on a number of inflammatory markers in a murine model of OVA-induced allergic airway inflammation. Following intranasal administration to mice, concentrations remained at least 100-fold higher in lung tissue than in plasma where low concentrations of POL7085 were detected at 24 h after dosing. POL7085 reduced AHR, bronchial inflammation, and bronchial remodelling in terms of collagen production, observations similar to those reported for CCL28 anti-serum in a virus challenge model [2].

CCR10 is the receptor for CCL28 chemokine, which is secreted by bronchial epithelium [9, 10]. In addition, high levels of CCL28 mRNA have been reported in human eosinophils [9]. We show here that lung levels of CCL28 protein were significantly increased after allergen challenge in OVA-sensitized mice. This finding is in accordance with reports showing increased CCL28 expression by airway epithelium and infiltrating leukocytes following antigen challenge in OVA- or cockroach-sensitized mice [1, 2]. In contrast, we observed variable BAL CCL28 mRNA levels, which is consistent with the report by English and collaborators that in cultured epithelial cells, expression of chemokine mRNA does not always correlate well with protein production and secretion [1]. Interestingly, we demonstrate that increased CCL28 lung levels induced by allergen challenge was totally abolished in mice treated with the CCR10 antagonist POL7085, whereas the glucocorticoid DEX had no effect. However, both agents markedly decreased the number of eosinophils and lymphocytes recruited to the airways. We therefore suggest that the contribution of eosinophils to increased lung CCL28 following allergen challenge is minor and most CCL28 production is likely by the airway epithelium, and/or that production of CCL28 by the infiltrated eosinophils is decreased by the CCR10 antagonist, and not by DEX. Additional *in vitro* experiments on airway epithelia cultured under air-liquid interface conditions would be required to further strengthen the dissociation between the inhibitory pathways induced by glucocorticoids and CCR10 antagonists in this asthma model.

Previously it has been reported that CCR10 expression was evident in both airway epithelium and infiltrating eosinophils following allergen challenge in a mouse model of OVA-induced allergic inflammation [1]. Here we confirm these findings using CCR10 immunolabelling, and in addition demonstrate CCR10 expression on endothelial and smooth muscle cells of the airways and pulmonary vasculature. Furthermore, in mice treated with the CCR10 antagonist POL7085, like the reference compound DEX, infiltrating inflammatory cells expressing CCR10 were clearly reduced.

Our data also show POL7085 reduced BAL eosinophil recruitment. This supports the observation that treatment with CCL28 anti-serum decreased airway eosinophil numbers [2]. These authors proposed CCL28 induces eosinophil chemotaxis through CCR3, but not CCR10 [2]. However, we observed that the CCR10 antagonist POL7085 markedly reduced IL-5 levels in lung homogenate and to a lesser extent in BAL fluid. In contrast, John and collaborators reported CCL28 anti-serum had only a weak inhibitory effect on Th2 cytokines or eosinophil-attracting chemokine levels in lung homogenate [2]. These contrasting data may be explained by differences between the models used such as single allergen challenge used by John and colleagues *versus* the multiple allergen challenges in our current study. Our data suggest CCL28 stimulates Th2 cells to increase IL-5 release and consequently eosinophil recruitment.

In addition, we report here the significant decrease in OVA-induced IL-13 production by POL7085. This decrease was not related to any decrease in mucus production in the airways by POL7085, neither in mucus staining of airway epithelium, nor in the mucus levels in whole lung, suggesting mucus secretion is under the dependence of other factors than IL-13 in this model. In addition, we show that IL-4 was significantly and dose-dependently decreased after treatment with POL7085, whereas there were no differences between treatment groups for OVA-specific IgE, indicating an effect of CCR10 antagonist on Th2 cells rather than a suppressive activity on IgE-secreting B cells.

These findings are consistent with the report that cross-linking of the FcεRI receptors expressed on lung conventional dendritic cells results in production of CCL28-mediated recruitment of IL-13-producing T cells induced by administration of Sendai virus to the lung, which was reduced by an anti-CCL28 antibody [22]. Viral infections are the most frequent causes of asthma exacerbations [23, 24] and severe viral infections during infancy may influence the development of post-viral atopic diseases via the conventional dendritic cell FcεRI-CCL28 pathway [25]. We thereby suggest that a study of the role of a CCR10 antagonist such as POL7085 in the CCL28-driven lung T cell recruitment during viral infections in mice would be useful.

During respiratory tract infections, in addition to its chemokine role in attracting immune cells expressing CCR10, CCL28 likely provides additional mucosal immunity by exerting an antimicrobial effect against pathogens [11, 14]. It is therefore important that any therapeutic agent targeting CCR10 in the airways does not interfere with this activity, particularly if it were administered in combination with immunosuppressive inhaled corticosteroids. To address this potential risk, we confirmed that POL7085 did not reduce the antimicrobial effect of CCL28 against *Pseudomonas aeruginosa* and *Candida albicans* in viable count assays [see Additional file 2].

In summary, here we present new evidence supporting a role for a CCR10 antagonist in allergen-induced bronchial inflammation, remodelling and AHR. Combined with previous findings, our data further implicate CCR10 in airway inflammatory diseases such as asthma. However, we need to be cautious when translating these data to the clinical setting, as exemplified by the recent report that despite strong evidence in preclinical models supporting a role for CCR3 in asthmatic disease [26], an oral CCR3 antagonist had limited efficacy in a randomized controlled clinical trial in asthmatics [27]. Nevertheless, our data give strong support for further investigations with CCR10 antagonists in the asthmatic disease, to allow clinical trials in particular in poorly controlled asthmatics.

## Additional files

Additional file 1:
**POL7085 selectivity profile. Inhibition of responses of a panel of GPCRs, ion channels and enzymes by POL7085.** Values are % inhibition produced by POL7085 at a single concentration of (5 or 10 μM), or IC_50_ where a full concentration-response was generated. Additional file 2:
**Antimicrobial effect of CCL28.** Figures show the effect of CCL28 on viability of *Pseudomonas aeruginosa* and *Candida albicans*, and that POL7085 does not interfere with this activity.

## References

[1] English K, Brady C, Corcoran P, Cassidy JP, Mahon BP (2006). Inflammation of the respiratory tract is associated with CCL28 and CCR10 expression in a murine model of allergic asthma. Immunol Lett.

[2] John AE, Thomas MS, Berlin AA, Lukacs NW (2005). Temporal production of CCL28 corresponds to eosinophil accumulation and airway hyperreactivity in allergic airway inflammation. Am J Pathol.

[3] O’Gorman MT, Jatoi NA, Lane SJ, Mahon BP (2005). Il-1βand TNFα induce increased expression of CCL28 by airway epithelial cells via an NFkB-dependent pathway. Cell Immunol.

[4] Holtzman MJ, Byers DE, Benoit LA, Battaile JT, You Y, Agapov E (2009). Immune pathways for translating viral infection into chronic airway disease. Adv Immunol.

[5] Jarmin DI, Rits M, Bota D, Gerard NP, Graham GJ, Clark-Lewis I (2000). Identification of the orphan receptor G-protein-coupled receptor 2 as CCR10, a specific receptor for the chemokine ESkine. J Immunol.

[6] Homey B, Wang W, Soto H, Buchanan ME, Wiesenborn A, Catron D (2000). The orphan chemokine receptor G protein-coupled receptor-2 (GPR2, CCR10) binds the skin-associated chemokine CCL27 (CTACK/ALP/ILC). J Immunol.

[7] Kunkel EJ, Kim CH, Lazarus NH, Vierra MA, Soler D, Bowman EP (2003). CCR10 expression is a common feature of circulating and mucosal epithelial tissue IgA Ab-secreting cells. J Clin Invest.

[8] Xiong N, Fu Y, Hu S, Xia M, Yang J (2012). CCR10 and its ligands in regulation of epithelial immunity and diseases. Protein Cell.

[9] Wang W, Soto H, Oldham ER, Buchanan ME, Homey B, Catron D (2000). Identification of a novel chemokine (CCL28), which binds CCR10 (GPR2). J Biol Chem.

[10] Pan J, Kunkel EJ, Gosslar U, Lazarus N, Langdon P, Broadwell K (2000). A novel chemokine ligand for CCR10 and CCR3 expressed by epithelial cells in mucosal tissues. J Immunol.

[11] Hieshima K, Ohtani H, Shibano M, Izawa D, Nakayama T, Kawasaki Y (2003). CCL28 has dual roles in mucosal immunity as a chemokine with broad-spectrum antimicrobial activity. J Immunol.

[12] Homey B, Alenius H, Müller A, Soto H, Bowman EP, Yuan W (2002). CCL27-CCR10 interactions regulate T cell-mediated skin inflammation. Nat Med.

[13] Scanlon KM, Hawksworth RJ, Lane SJ, Mahon BP (2011). IL-17A induces CCL28, supporting the chemotaxis of IgE-secreting B cells. Int Arch Allergy Immunol.

[14] Berri M, Virlogeux-Payant I, Chevaleyre C, Melo S, Zanello G, Salmon H (2014). CCL28 involvement in mucosal tissues protection as a chemokine and as an antibacterial peptide. Dev Comp Immunol.

[15] Alvarez D, Arkinson JL, Sun J, Fattouh R, Walker T, Jordana M (2007). Th2 differentiation in distinct lymph nodes influences the site of mucosal Th2 immune-inflammatory response. J Immunol.

[16] Robinson JA, DeMarco S, Gombert F, Moehle K, Obrecht D (2008). The design, structures and therapeutic potential of protein epitope mimetics. Drug Discov Today.

[17] Jung F, Gombert FO, Obrecht D, Bisang C, Barthelemy S, Lederer A, et al. Template-fixed peptidomimetics with CCR10 antagonistic activity. WO/2011/060937.

[18] Daubeuf F, Frossard N (2013). Acute Ovalbumin asthma model in the mouse. Curr Protoc Mouse Biol.

[19] Hamelmann E, Schwarze J, Takeda K, Oshiba A, Larsen LG, Irvin CG (1997). Noninvasive measurement of airway responsiveness in allergic mice using barometric plethysmography. Am J Respir Crit Care Med.

[20] Daubeuf F, Reber L, Frossard N (2013). Measurement of airway responsiveness on vigil and unrestrained mouse. Bio-protocols.

[21] Daubeuf F, Frossard N (2012). Performing bronchoalveolar lavage in the mouse. Curr Protoc Mouse Biol.

[22] Grayson MH, Cheung D, Rohlfing MM, Kitchens R, Spiegel DE, Tucker J (2007). Induction of high-affinity IgE receptor on lung dendritic cells during viral infection leads to mucous cell metaplasia. J Exp Med.

[23] Busse WW, Lemanske RF, Gern JE (2010). The role of viral respiratory infections in asthma and asthma exacerbations. Lancet.

[24] Jackson DJ, Sykes A, Mallia P, Johnston SL (2011). Asthma exacerbations: origin, effect, and prevention. J Allergy Clin Immunol.

[25] Tam JS, Grayson MH. Dendritic cells, viruses, and the development of atopic disease. J Allergy. 2012;2012: Article ID 936870. 10.1155/2012/936870 PMC3478734 23118777

[26] Wegmann M (2011). Targeting eosinophil biology in asthma therapy. Am Resp Cell Mol Biol.

[27] Neighbour H, Boulet L-P, Lemiere C, Sehmi R, Leigh R, Sousa AR (2014). Safety and efficacy of an oral CCR3 antagonist in patients with asthma and eosinophilic bronchitis: a randomized, placebo-controlled clinical trial. Clin Exp Allergy.

