# Supplementary material for: Protective effect of a Protein Epitope Mimetic CCR10 antagonist, POL7085, in a model of allergic eosinophilic airway inflammation
Source: Respir Res. 2015 Jun 27;16(1):77. doi: 10.1186/s12931-015-0231-5 (PMC4490744; doi:10.1186/s12931-015-0231-5)
Supplement: Additional file 1: — POL7085 selectivity profile. Inhibition of responses of a panel of GPCRs, ion channels and enzymes by POL7085. Values are % inhibition produced by POL7085 at a single concentration of (5 or 10 μM), or IC50 where a full concentration-response was generated. [file 12931_2015_231_MOESM1_ESM.pdf]

Receptor	Species	Assay	Parameter	Value
5-HT2A	human	Binding	Inh(%) [10 µM]	12
Adrenergic alpha1A	rat	Binding	Inh(%) [10 µM]	37
Adrenergic alpha1D	human	Binding	Inh(%) [10 µM]	2
Adrenergic beta1	human	Binding	Inh(%) [10 µM]	-8
Angiotensin 2	human	Binding	Inh(%) [10 µM]	39
Apelin	human	B-arrestin	Inh(%) [10 µM]	14
Bradykinin B1	human	Binding	Inh(%) [10 µM]	19
Bradykinin B2	human	Binding	Inh(%) [10 µM]	56
CB1	human	Binding	Inh(%) [10 µM]	28
CB2	human	Binding	Inh(%) [10 µM]	-6
CCR1	human	Calcium Flux	Inh(%) [10 µM]	-6
CCR2	human	Calcium Flux	Inh(%) [10 µM]	0
CCR3	human	Calcium Flux	Inh(%) [10 µM]	32
CCR4	human	Calcium Flux	Inh(%) [10 µM]	21
CCR5	human	Calcium Flux	Inh(%) [10 µM]	0
CCR6	human	Calcium Flux	Inh(%) [10 µM]	27
CCR7	human	Calcium Flux	Inh(%) [10 µM]	-47
CCR8	human	Calcium Flux	Inh(%) [10 µM]	-20
CCR9	human	Calcium Flux	Inh(%) [10 µM]	0
CCR10	human	Calcium Flux	IC50 [nM]	42
CGRP1	human	Binding	Inh(%) [10 µM]	-19
CRF1	human	Binding	Inh(%) [10 µM]	7
CRTH2	human	Binding	Inh(%) [10 µM]	4
CX3CR1	human	Calcium Flux	Inh(%) [10 µM]	61
CXCR1	human	Calcium Flux	Inh(%) [10 µM]	36
CXCR2	human	Calcium Flux	Inh(%) [10 µM]	53
CXCR3	human	Calcium Flux	Inh(%) [10 µM]	-11
CXCR4	human	Calcium Flux	Inh(%) [10 µM]	27
CXCR6	human	Calcium Flux	Inh(%) [10 µM]	24
CXCR7	human	Calcium Flux	Inh(%) [10 µM]	44
Dopamine receptor D2	human	Binding	Inh(%) [10 µM]	4
Endothelin ETa	human	Binding	Inh(%) [10 µM]	5
Endothelin ETb	human	Binding	Inh(%) [10 µM]	1
F2RL1 (PAR2)	human	B-arrestin	Inh(%) [10 µM]	-21
FPR1	human	B-arrestin	Inh(%) [10 µM]	2
FPR2	human	B-arrestin	Inh(%) [10 µM]	-11
Histamine H1	human	Binding	Inh(%) [10 µM]	-7
LTB4 receptor	human	Binding	Inh(%) [10 µM]	-3
Muscarinic M3	human	Binding	Inh(%) [10 µM]	-7
NK1	human	Binding	Inh(%) [10 µM]	6
Opiate (OP3)	human	Binding	Inh(%) [10 µM]	-4
Prostanoid EP2	human	Binding	Inh(%) [10 µM]	15
Purinergic P2Y	rat	Binding	Inh(%) [10 µM]	10
Somatostatin sst1	human	Binding	Inh(%) [10 µM]	40
VPAC1	human	Binding	Inh(%) [10 µM]	-4
Cav2.2	human	Calcium Flux	Inh(%) [5 µM]	4
Kv1.3	human	Thallium Flux	Inh(%) [5 µM]	-6
Nav1.7	human	Thallium Flux	Inh(%) [5 µM]	2
Nicotinic acetylcholine receptor	human	Binding	Inh(%) [10 µM]	-3
P2X2/3	human	Calcium Flux	Inh(%) [5 µM]	7
P2X7	human	Calcium Flux	Inh(%) [10 µM]	-4
Potassium channel hERG	human	Automated Patch Clamp	IC50 (M)	> 1.5E-04
Potassium channel Skca	rat	Binding	Inh(%) [10 µM]	-10
IL-1 receptor	mouse	Binding	Inh(%) [10 µM]	-6
IL-6 receptor	human	Binding	Inh(%) [10 µM]	2
Cathepsin C	human	Enzymatic assay	Inh(%) [10 µM]	5
MMP1	human	Enzymatic assay	Inh(%) [10 µM]	4
MMP9	human	Enzymatic assay	Inh(%) [10 µM]	15
