# Supplementary figures and images for: Protective effect of a Protein Epitope Mimetic CCR10 antagonist, POL7085, in a model of allergic eosinophilic airway inflammation
Source: Respir Res. 2015 Jun 27;16(1):77. doi: 10.1186/s12931-015-0231-5 (PMC4490744; doi:10.1186/s12931-015-0231-5)

## Effect of POL7085 on antimicrobial activity of CCL28

(a) *Pseudomonas aeruginosa*

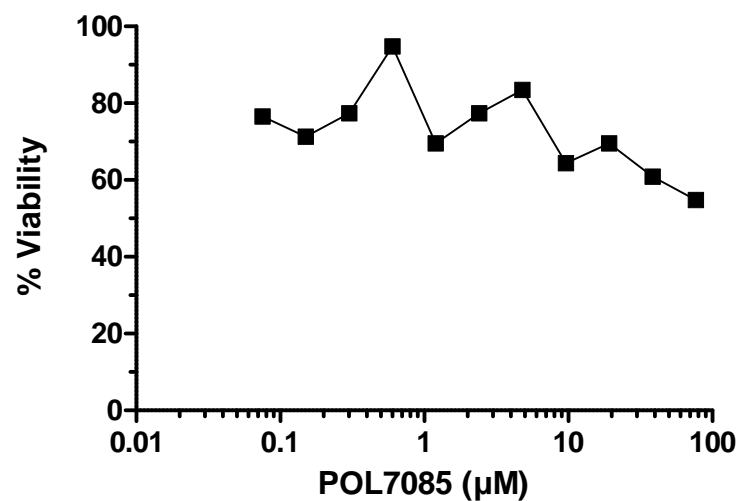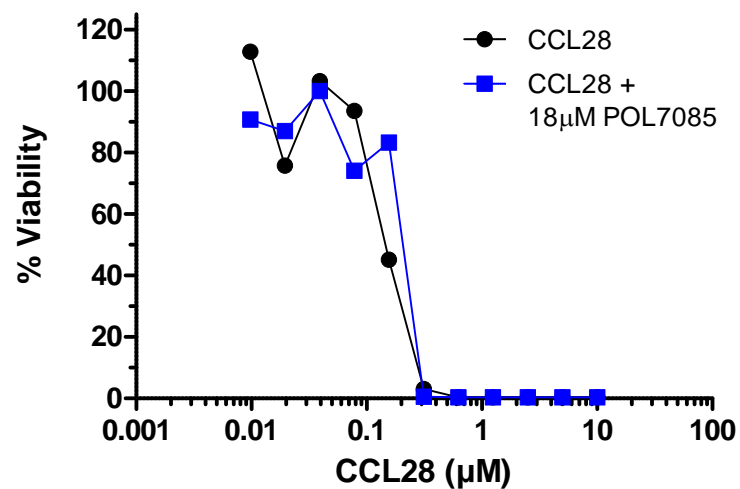

(b) *Candida albicans*

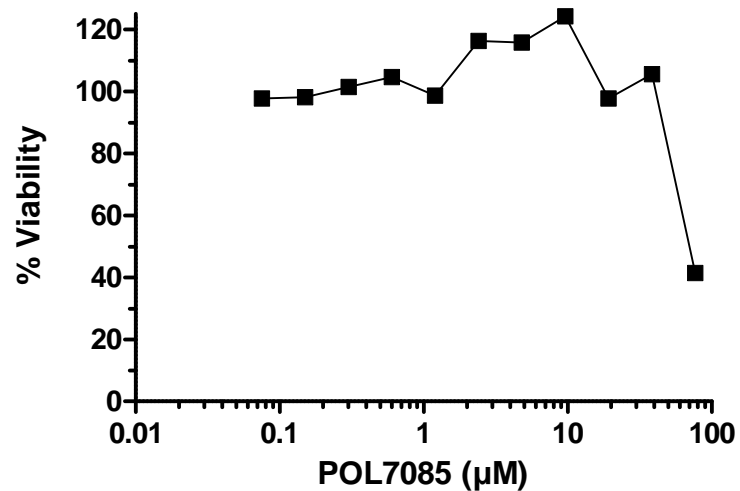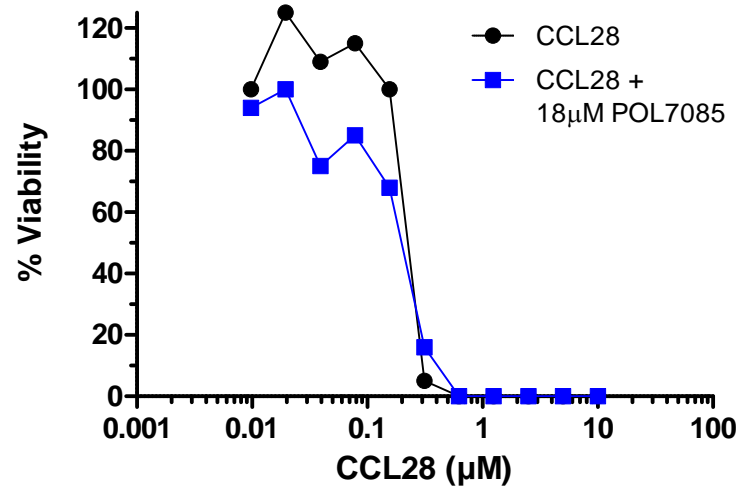

Supplement: Additional file 2: — Antimicrobial effect of CCL28. Figures show the effect of CCL28 on viability of Pseudomonas aeruginosa and Candida albicans, and that POL7085 does not interfere with this activity. [file 12931_2015_231_MOESM2_ESM.pdf]
